# Sarcopenia reduces quality of life in the long-term: longitudinal analyses from the English longitudinal study of ageing

**DOI:** 10.1007/s41999-022-00627-3

**Published:** 2022-02-25

**Authors:** Nicola Veronese, Ai Koyanagi, Emanuele Cereda, Stefania Maggi, Mario Barbagallo, Ligia J. Dominguez, Lee Smith

**Affiliations:** 1 Geriatric Unit, Department of Internal Medicine and Geriatrics, University of Palermo, via del Vespro, 141, 90127 Palermo, Italy; 2 Research and Development Unit, Parc Sanitari Sant Joan de Déu, CIBERSAM, Dr. Antoni Pujadas, 42, 08830 Sant Boi de Llobregat, Barcelona Spain; 3 ICREA, Pg, Lluis Companys 23, 08010 Barcelona, Spain; 4 Clinical Nutrition and Dietetics Unit, Fondazione IRCCS Policlinico San Matteo, Pavia, Italy; 5 Institute of Neuroscience, National Research Council, Padova, Italy; 6 Centre for Health, Performance and Wellbeing, Anglia Ruskin University, Cambridge, UK

**Keywords:** Quality of life, Sarcopenia, ELSA, Older adults, Epidemiology, Longitudinal, Ageing

## Abstract

**Purpose:**

Mixed findings exist for sarcopenia/quality of life (QoL) relationship. Moreover, the majority of studies in this area have utilized a cross-sectional design or specific clinical populations. Therefore, the aim of the present study was to examine the association between sarcopenia at baseline and QoL at 10 years follow-up in a large representative sample of older English adults.

**Methods:**

Sarcopenia was diagnosed as having low handgrip strength and low skeletal muscle mass index. QoL was measured using the CASP (control, autonomy, self-realisation and pleasure)-19, with higher values reflecting higher QoL. Multivariable logistic regression analysis was conducted to assess prospective associations between sarcopenia at baseline and poor QoL at follow-up; generalized linear model with repeated measures was used for reporting mean changes during follow-up between sarcopenia and not.

**Results:**

Among 4044 older participants initially included at baseline (mean age: 70.7 years; 55.1% females), 376 had sarcopenia. In the multivariable analysis, after adjusting for several potential confounders, sarcopenia at baseline was associated with a higher incidence of poor QoL (odds ratio, OR = 5.82; 95% confidence interval, CI 3.45–9.82). After matching for QoL values at baseline and adjusting for potential confounders, people with sarcopenia reported significantly lower values in CASP-19 (mean difference = − 3.94; 95% CI − 4.77 to − 3.10).

**Conclusions:**

In this large representative sample of older English adults, it was observed that sarcopenia at baseline was associated with worse scores of QoL at follow-up compared to those without sarcopenia at baseline. It may be prudent to target those with sarcopenia to improve QoL.

## Key summary points

**Aim:**

To examine the association between sarcopenia at baseline and changes in quality of life at 10 years follow-up in a large representative sample of older English adults.

**Findings:**

After considering numerous confounders, sarcopenia at baseline was associated with a higher incidence of poor quality of life. People having sarcopenia at baseline reported significantly lower values in CASP-19 after ten years of follow-up.

**Message:**

Sarcopenia is an important and independent risk factor for poor quality of life in older people.

## Background

Quality of life (QoL) is one’s perception of their position in life in the context of the culture and value systems in which one lives, and in relation to one’s goals, expectations, standards and concerns [1]. QoL is indeed an important measure of overall health, including wellbeing [2], Health organizations emphasize that maintaining good QoL is of importance throughout the life course [2].

Importantly, QoL has been observed to be associated with mortality. For example, in one review of several studies, including approximately 1,200,000 participants, it was found that better QoL was associated with lower mortality risk [3]. This association has been found to be strong in older adults particularly when health-related QoL is used as an outcome. For example, in a prospective cohort study of 2373 persons, representative of the Spanish population aged 60 and older, it was found that changes in health-related QoL predicted mortality [4]. It is thus important to identify correlates of QoL in older adults to inform targeted interventions to improve QoL or maintain adequate levels. One potential but understudied correlate of QoL in older adults is that of sarcopenia.

Sarcopenia refers to “age-related muscle loss, affecting a combination of appendicular muscle mass, muscle strength, and/or physical performance measures” [5] and is now widely considered to be a disease [6]. Sarcopenia is plausibly linked to a reduction in QoL as it has been observed to be associated with higher rates of hospitalization, dependency, falls and disability [7]. In one systematic review, 11 studies investigating QoL in sarcopenic people were identified, and interestingly, the results were quite heterogenous showing either no difference in QoL between sarcopenic and non-sarcopenic participants or poorer QoL for sarcopenic patients, but generally only for specific QoL domains [8]. At the same time, generic questionnaires are used for measuring QoL in sarcopenia and this can further limit the literature available about the potential associations between sarcopenia and QoL [8]. Importantly, only two of the included studies were longitudinal in nature. In one study consisting of 670 older adults from Taiwan, it was observed that sarcopenia at baseline was associated with worse QoL scores at 4-year follow-up [9]. The other longitudinal study consisted of a sample of 230 patients listed for liver transplant and found no association between baseline sarcopenia and QoL at follow-up [10]. Other more recent studies, and thus not include in the review, have investigated the sarcopenia/QoL relationship in those with specific medical conditions e.g. dementia [11] or cancer [12]. It is clear that further research of a longitudinal nature is needed, with a longer follow-up, in large representative samples of the general older adult population to further elucidate the association between sarcopenia and QoL.

Given this background, the aim of the present study was to examine the association between sarcopenia at baseline and QoL at 10-year follow-up in a large representative sample of older English adults.

## Methods

### Study population

This study is based on data from six waves (from Wave 2 to Wave 7) of the English Longitudinal Study of Ageing (ELSA), which is a prospective and nationally representative cohort of men and women living in England [13]. Wave 2 (baseline survey) was conducted in 2004–2005; the other waves were conducted every 2 years, until Wave 7 between 2014 and 2015. The ELSA study was approved by the London Multicentre Research Ethics Committee (MREC/01/2/91). Informed consent was obtained from all participants.

### Sarcopenia (independent variable)

Following the criteria of the revised European consensus on the definition and diagnosis of sarcopenia [14], sarcopenia was defined as weak handgrip strength and having low skeletal muscle mass (SMM), as reflected by lower skeletal mass index (SMI). Briefly, SMM was calculated based on the equation proposed by Lee and colleagues[15]:

SMM = 0.244*weight + 7.8*height + 6.6*sex – 0.098*age + race – 3.3

(where female = 0 and male = 1; race = 0 [White and Hispanic], race = 1.4 [Black] and race = − 1.2 [Asian]).

SMM was further divided by body mass index (BMI) based on weight and height measured by a trained nurse, to create a SMI [16]. Low SMM was defined as the lowest quartile of the SMI based, on sex-stratified values [17]. Weak handgrip strength was defined as < 27 kg for men and < 16 kg for women using the average value of three handgrip measurements of the dominant hand [14]. Grip strength in kilograms was measured by using a Smedley dynamometer (TTM; Tokyo, Japan), with the upper arm being held against the trunk and the elbow in a 90-degree flexion [13]. We also investigated the onset of sarcopenia during the follow-up waves.

### Outcomes: quality of life

The QoL measure used in the ELSA is CASP (control, autonomy, self-realisation and pleasure)-19 [18]. It is a self-completion questionnaire and spans four derived dimensions based on Likert scaled items. CASP-19 has an overall summary measure on a 0–57 scale, with higher scores corresponding to greater well-being [18]. CASP-19 was evaluated in each wave included in this analysis, from wave 2 to 7. Poor QoL was defined, arbitrarily, as values falling in the lowest quartile values of CASP-19 in each wave.

### Covariates

The selection of covariates was based on their previously reported associations with the exposure (sarcopenia) and outcome (poor QoL), and included the following: age; sex; years of education (considered as continuous variable); ethnicity (whites vs. non-whites); marital status (married vs. other status); smoking status (ever vs. never); physical activity level (high vs. moderate/low/sedentary): in the ELSA study, for assessing physical activity level, three questions were made regarding vigorous, moderate, mild activity in the previous twelve months. To assist in answering the questions, prompt cards with examples of activities categorised by intensity [19]; the presence of depressive symptoms assessed with the Center for Epidemiologic Studies Depression Scale (CES-D) [20]; the presence of multimorbidity was defined as ≥ 2 chronic conditions [21], among all the medical conditions assessed at wave 2. All these parameters were assessed at baseline.

### Statistical analyses

The data were weighted using the person-level longitudinal weight, core sample, wave 2 (http://www.ifs.org.uk/ELSA). The analysis was restricted to those aged ≥ 60 years at baseline as sarcopenia is an age-related condition.

Continuous variables were analyzed in terms of distribution using the Kolmogorov–Smirnov test, using the Levene’s test to test the homoscedasticity of variances. Means and standard deviations (SD) were used to describe quantitative measures, while percentages and counts were used for categorical variables. Characteristics of the study participants at baseline (wave 2) were compared according to the presence of sarcopenia at wave 2 with the use of Chi-squared or Fisher exact tests for categorical variables, and independent *t* test for continuous variables.

The association between sarcopenia at baseline and poor quality of life during follow-up was assessed using univariable and multivariable logistic regression analysis and reported as odds ratios (OR) and 95% confidence intervals (95% CI). Participants falling in the lowest quartile of CASP-19 at the baseline evaluation were removed from this analysis. Moreover, the association between sarcopenia at the baseline and the changes of CASP-19 during follow-up was evaluated using a generalized linear model with repeated measures, adjusting for the potential confounders mentioned in the covariates paragraph, including CASP-19 values at baseline. For missing data during follow-up regarding CASP-19, a multiple imputation method was used, with a maximum of 20 interactions [22]. For further minimizing the possible bias, we used a case–control match (1:10) using similar values of CASP-19 at wave 2 between people with and without sarcopenia.

All statistical tests were two-tailed, and a *p* value < 0.05 was considered to be statistically significant. All analyses were performed using SPSS 21.0 version software.

## Results

**Fig. 1:**
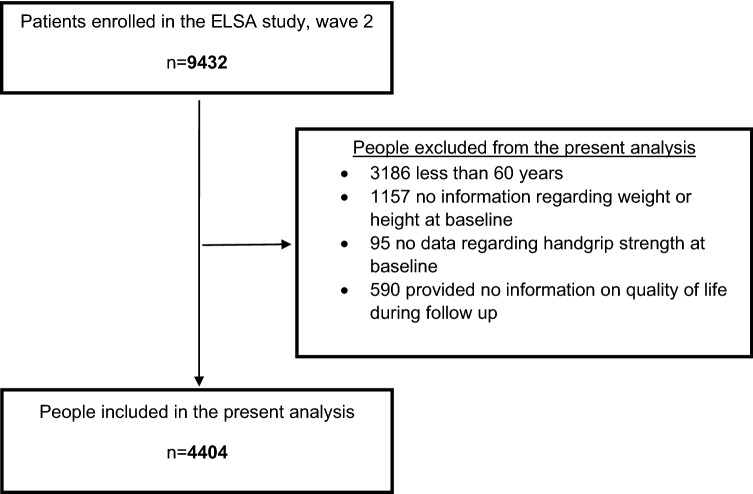
Flowchart of the selection of participants

A total of 9432 participants were included in the second wave (baseline) of the ELSA. In the present analyses 3186 participants were excluded owing to having an age < 60 years, 1157 owing to missing wave 2 data on body composition, 95 owing to missing wave 2 data on handgrip strength, and 590 owing to missing data on QoL at follow-up. The present analytic study population included 4044 participants (Fig. 1, unweighted data).

Participants mean age was 70.7 ± SD 7.6 years (range 60–90) and 55.1% were female. The baseline prevalence of sarcopenia was 8.5%. Table 1 shows the main descriptive characteristics of the participants included, according to the presence or absence of sarcopenia at wave 2. Those who were sarcopenic at baseline (*n* = 376) were significantly older, more likely to be male, not married, and had a lower level of education compared to those without sarcopenia (*n* = 4028) (all = *p* < 0.0001). People with sarcopenia were less physically active, more depressed, and had worse CASP-19 scores, at baseline (*p* < 0.0001) (Table 1).

**Table 1:** Baseline characteristics by presence or absence of sarcopenia

Parameter	Sarcopenia (*n* = 376)	No sarcopenia (*n* = 4028)	*p *value
Age (mean, SD)	79.7 (7.4)	69.8 (7.0)	< 0.0001
Female gender (*n*, %)	147 (39.1)	1829 (45.4)	0.02
Years of education (mean, SD)	3.4 (5.8)	6.7 (6.9)	< 0.0001
Whites (*n*, %)	363 (96.5)	3977 (98.7)	0.002
Married (*n*, %)	153 (40.7)	2564 (65.9)	< 0.0001
Ever smoked (*n*, %)	236 (62.8)	2530 (62.8)	1.00
High physical activity level (*n*, %)	22 (5.9)	726 (18.09	< 0.0001
CASP-19 (mean, SD)	31.3 (17.3)	35.8 (11.7)	< 0.0001
CES-D (mean, SD)	2.22 (1.99)	1.43 (1.83)	< 0.0001
Multimorbidity (n, %)	309 (82.2)	2568 (63.8)	< 0.0001

*SD* standard deviation, *CASP-19* control, autonomy, self-realisation and pleasure-19, *CES-D* center for epidemiologic studies depression scale

Table 2 shows the association between sarcopenia at baseline and the onset of poor QoL. In the univariable analysis, sarcopenia at baseline was associated with a higher incidence of poor QoL (OR = 10.14; 95% CI 6.29–16.35; *p* < 0.0001): this association remained statistically significant after adjusting for potentially important confounding variables (OR = 5.82; 95% CI 3.45–9.82; *p* < 0.0001) (Table 2).

**Table 2:** Association between sarcopenia at the baseline and incident poor quality of life (weighted data)

	Univariable model (OR, 95% CI)	*p*-value	Fully-adjusted model^a^ (OR, 95% CI)	*p*-value
No sarcopenia	Reference	–	Reference	–
Sarcopenia	10.14 (6.29–16.35)	< 0.0001	5.82 (3.45–9.82)	< 0.0001

Data are reported as odds ratios (ORs) with their 95% confidence intervals (CIs)

Incident poor quality of life was defined as the lowest quartile in control, autonomy, self-realisation and pleasure-19 values, specific for each wave

^a^Fully-adjusted model included: age (as continuous variable); gender; years of education (as continuous variable); ethnicity (whites vs. non-whites); marital status (married vs. other status); smoking status (ever vs. never); Center for Epidemiologic Studies Depression Scale (as continuous variable); physical activity level (high vs. others); presence of multimorbidity (yes vs. no); control, autonomy, self-realisation and pleasure-19 values at baseline; changes in sarcopenia status during follow-up period

**Fig. 2:**
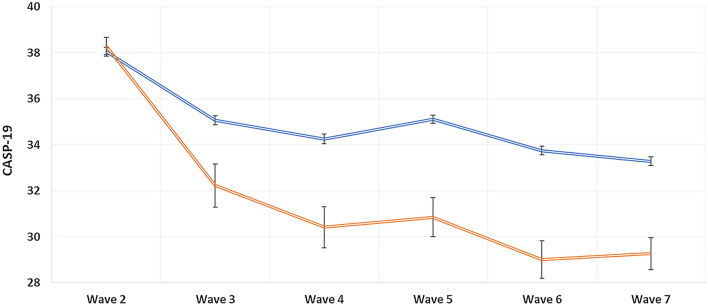
Changes of quality of life, during follow-up, by presence of sarcopenia or not at baseline (weighted data). Data are reported as mean and standard errors by presence of sarcopenia (red line) and absence of this condition (blue line), matching people with and without sarcopenia for CASP-19 values at wave 2. Analyses were adjusted for age (as continuous variable); gender; years of education (as continuous variable); ethnicity (whites vs. non-whites); marital status (married vs. other status); smoking status (ever vs. never); Center for Epidemiologic Studies Depression Scale (as continuous variable); physical activity level (high vs. others); presence of multimorbidity (yes vs. no)

Figure 2 shows the association between those with sarcopenia (*n* = 269) and thosewithout sarcopenia at baseline (*n* = 2690) in terms of QoL during follow-up, after baseline matching of CASP-19 scores. After 10 years of follow-up, people with sarcopenia reported significantly lower values in CASP-19 (mean difference = − 3.94; 95% CI − 4.77 to − 3.10; *p* < 0.0001), after adjusting for potentially important confounding variables.

## Discussion

In the present large representative sample of older adults with 10 years of follow-up, it was observed that the presence of sarcopenia at baseline was significantly associated with lower scores of QoL at follow-up compared to those who were not sarcopenic.

The present findings support and add to the only other longitudinal analyses carried out in a small sample (*n* = 670) of Taiwanese older adults and observed that sarcopenia at baseline was associated with worse QoL scores at 4-year follow-up [9]. It adds to this study through demonstrating that such an association holds in a large sample (4044) of older English adults with 10 years of follow-up. There are several plausible pathways that likely explain the association between sarcopenia and worse QoL scores. First, sarcopenia is associated with a greater risk of functional impairment and disability, which may limit one’s ability to carry out certain functions in their daily lives [23] potentially impacting their QoL [24]. Second, sarcopenia is associated with higher risk of falling [25] and, therefore, may experience fractures owing to such falls or avoidance of specific behaviors owing to fear of falling, both of which may limit activities of daily life and thus reduce QoL [25]. Third, sarcopenia has been observed to be associated with multiple mental health complications [26] and mental health complications are indeed associated with a worsening of QoL [27]. Fourth, sarcopenia has been found to reduce sleep quality [28] potentially owing to a low level of irisin [29]. Poor sleep likely worsens QoL owing to excessive daytime sleepiness and lack of energy.

The findings from the present study and the only other longitudinal study in a sample of “healthy” older adults [9] suggest that it may be prudent for interventions to improve QoL to be targeted at those with sarcopenia. It may be beneficial to incorporate mind–body exercises into such interventions such as Tai-chi. Indeed, literature has shown that such exercises can significantly reduce sarcopenia symptoms [30] and improve QoL per se [31].

The large representative sample and 10-year follow-up are strengths of the present study. However, findings must be interpreted in light of the studies limitations. Just one measure of muscle strength was employed to measure sarcopenia. Although a unified geriatric assessment tool has not as of yet been widely utilized to assess for sarcopenia, handgrip strength is indeed frequently implemented to measure muscle strength, which isa critical component of sarcopenia [14]. Handgrip strength is commonly employed in both research and clinical practice [32] and has been found to be an independent predictor of early mortality [33]. Next, the body composition estimate was calculated utilising a population equation and not direct assessment. In this sense, this equation did not consider some common anthropometric measures, such as hip circumference and the proposed equation was validated only in the USA, whilst no direct evaluation was made in Europe and particularly in the ELSA study [15]. Third, the CASP-19 has not been validated in sarcopenic people. Future similar research should seek to implement the SarQoL [34], which is the only validated QOL tool for use in people with sarcopenia, unfortunately, not available at the time of the baseline assessment of the ELSA study. Finally, it would be interesting to explore the possible association between sarcopenia and the altered dimensions of QoL, but this important information is unfortunately not available in CASP-19 scale [35].

In conclusion, in this large representative sample of older English adults, it was observed that sarcopenia at baseline was associated with worse scores of QoL at follow-up compared to those without sarcopenia at baseline. It may be prudent to target those with sarcopenia to improve QoL.

## References

[1] Group W (1993). Study protocol for the World Health Organization project to develop a Quality of Life assessment instrument (WHOQOL). Qual Life Res.

[2] Group W (1995). The World Health Organization quality of life assessment (WHOQOL): position paper from the World Health Organization. Soc Sci Med.

[3] Phyo AZZ, Freak-Poli R, Craig H, Gasevic D, Stocks NP, Gonzalez-Chica DA, Ryan J (2020). Quality of life and mortality in the general population: a systematic review and meta-analysis. BMC Public Health.

[4] Otero-Rodríguez A, León-Muñoz LM, Balboa-Castillo T, Banegas JR, Rodríguez-Artalejo F, Guallar-Castillón P (2010). Change in health-related quality of life as a predictor of mortality in the older adults. Qual Life Res.

[5] Walston JD (2012). Sarcopenia in older adults. Curr Opin Rheumatol.

[6] Anker SD, Morley JE, von Haehling S (2016). Welcome to the ICD-10 code for sarcopenia. J Cachexia Sarcopenia Muscle.

[7] Woo J (2017). Sarcopenia. Clin Geriatr Med.

[8] Beaudart C, Reginster J-Y, Geerinck A, Locquet M, Bruyère O (2017). Current review of the SarQoL®: a health-related quality of life questionnaire specific to sarcopenia. Expert Rev Pharmacoecon Outcomes Res.

[9] Wu T-Y, Liaw C-K, Chen F-C, Kuo K-L, Chie W-C, Yang R-S (2016). Sarcopenia screened with SARC-F questionnaire is associated with quality of life and 4-year mortality. J Am Med Dir Assoc.

[10] Yadav A, Chang YH, Carpenter S, Silva AC, Rakela J, Aqel BA, Byrne TJ, Douglas DD, Vargas HE, Carey EJ (2015). Relationship between sarcopenia, six-minute walk distance and health-related quality of life in liver transplant candidates. Clin Transplant.

[11] Umegaki H, Bonfiglio V, Komiya H, Watanabe K, Kuzuya M (2020). Association between sarcopenia and quality of life in patients with early dementia and mild cognitive impairment. J Alzheimers Dis.

[12] Nipp RD, Fuchs G, El-Jawahri A, Mario J, Troschel FM, Greer JA, Gallagher ER, Jackson VA, Kambadakone A, Hong TS (2018). Sarcopenia is associated with quality of life and depression in patients with advanced cancer. Oncologist.

[13] Steptoe A, Breeze E, Banks J, Nazroo J (2013). Cohort profile: the English longitudinal study of ageing. Int J Epidemiol.

[14] Cruz-Jentoft AJ, Bahat G, Bauer J (2019). Sarcopenia: revised European consensus on definition and diagnosis. Age Ageing.

[15] Lee RC, Wang Z, Heo M, Ross R, Janssen I, Heymsfield SB (2000). Total-body skeletal muscle mass: development and cross-validation of anthropometric prediction models. Am J Clin Nutr.

[16] Studenski SA, Peters KW, Alley DE (2014). The FNIH sarcopenia project: rationale, study description, conference recommendations, and final estimates. J Gerontol A Biol Sci Med Sci.

[17] Tyrovolas S, Koyanagi A, Olaya B, Ayuso-Mateos JL, Miret M, Chatterji S, Tobiasz-Adamczyk B, Koskinen S, Leonardi M, Haro JM (2016). Factors associated with skeletal muscle mass, sarcopenia, and sarcopenic obesity in older adults: a multi-continent study. J Cachexia Sarcopenia Muscle.

[18] Howel D (2012). Interpreting and evaluating the CASP-19 quality of life measure in older people. Age Ageing.

[19] McMullan II, Bunting BP, McDonough SM, Tully MA, Casson K (2020). The association between light intensity physical activity with gait speed in older adults (≥ 50 years). A longitudinal analysis using the English Longitudinal Study of Ageing (ELSA). Aging Clin Exp Res.

[20] Eaton WW, Smith C, Ybarra M, Muntaner C, Tien A (2004) Center for Epidemiologic Studies Depression Scale: review and revision (CESD and CESD-R).

[21] Garin N, Koyanagi A, Chatterji S (2016). Global multimorbidity patterns: a cross-sectional, population-based, multi-country study. J Gerontol A Biol Sci Med Sci.

[22] Royston P (2004). Multiple imputation of missing values. Stand Genomic Sci.

[23] Janssen I, Heymsfield SB, Ross R (2002). Low relative skeletal muscle mass (sarcopenia) in older persons is associated with functional impairment and physical disability. J Am Geriatr Soc.

[24] Sahoo P, Sethy RR, Ram D (2017). Functional impairment and quality of life in patients with obsessive compulsive disorder. Indian J Psychol Med.

[25] Yeung SS, Reijnierse EM, Pham VK, Trappenburg MC, Lim WK, Meskers CG, Maier AB (2019). Sarcopenia and its association with falls and fractures in older adults: a systematic review and meta-analysis. J Cachexia Sarcopenia Muscle.

[26] Chang K-V, Hsu T-H, Wu W-T, Huang K-C, Han D-S (2017). Is sarcopenia associated with depression? A systematic review and meta-analysis of observational studies. Age Ageing.

[27] Brenes GA (2007). Anxiety, depression, and quality of life in primary care patients. Primary Care Compan J Clin Psychiatry.

[28] Nagaura Y, Kondo H, Nagayoshi M, Maeda T (2020). Sarcopenia is associated with insomnia in Japanese older adults: a cross-sectional study of data from the Nagasaki Islands study. BMC Geriatr.

[29] Park H-S, Kim HC, Zhang D, Yeom H, Lim S-K (2019). The novel myokine irisin: clinical implications and potential role as a biomarker for sarcopenia in postmenopausal women. Endocrine.

[30] Morawin B, Tylutka A, Chmielowiec J, Zembron-Lacny A (2021). Circulating mediators of apoptosis and inflammation in aging; physical exercise intervention. Int J Environ Res Public Health.

[31] Wang D, Wang P, Lan K, Zhang Y, Pan Y (2020). Effectiveness of Tai chi exercise on overall quality of life and its physical and psychological components among older adults: a systematic review and meta-analysis. Braz J Med Biol Res.

[32] Roberts HC, Denison HJ, Martin HJ, Patel HP, Syddall H, Cooper C, Sayer AA (2011). A review of the measurement of grip strength in clinical and epidemiological studies: towards a standardised approach. Age Ageing.

[33] Wu Y, Wang W, Liu T, Zhang D (2017). Association of grip strength with risk of all-cause mortality, cardiovascular diseases, and cancer in community-dwelling populations: a meta-analysis of prospective cohort studies. J Am Medical Direct Assoc.

[34] Beaudart C, Biver E, Reginster JY, Rizzoli R, Rolland Y, Bautmans I, Petermans J, Gillain S, Buckinx F, Dardenne N (2017). Validation of the SarQoL®, a specific health-related quality of life questionnaire for Sarcopenia. J Cachexia Sarcopenia Muscle.

[35] Veronese N, Smith L, Pizzol D, Soysal P, Maggi S, Ilie P-C, Dominguez LJ, Barbagallo M (2022) Urinary incontinence and quality of life: a longitudinal analysis from the English Longitudinal Study of Ageing. Maturitas 10.1016/j.maturitas.2022.01.010 35550703

